# Supplementary figures and images for: The complete mitochondrial genome of the grooved carpet shell, Ruditapes decussatus (Bivalvia, Veneridae)
Source: PeerJ. 2017 Aug 22;5:e3692. doi: 10.7717/peerj.3692 (PMC5571815; doi:10.7717/peerj.3692)

**Supplementary Figure 1- mitochondrial tRNA structures of *Ruditapes decussatus***

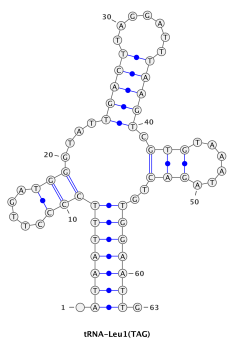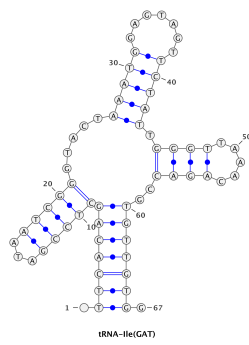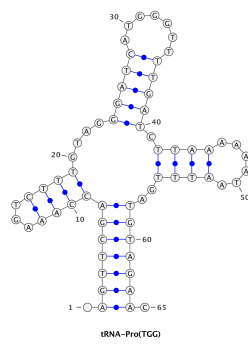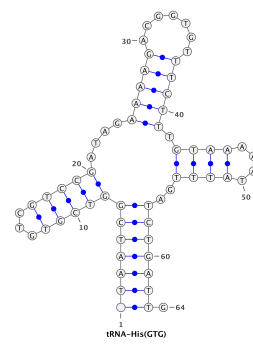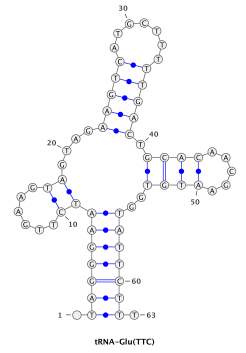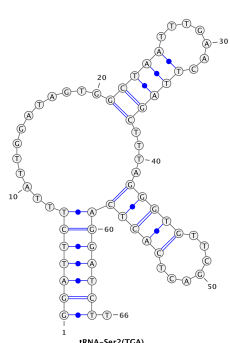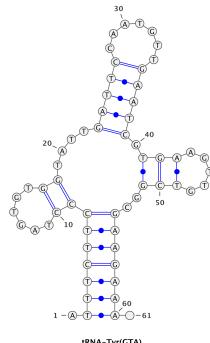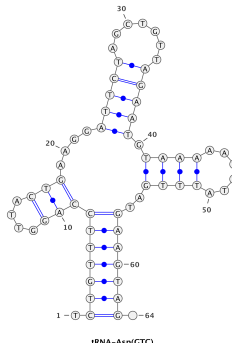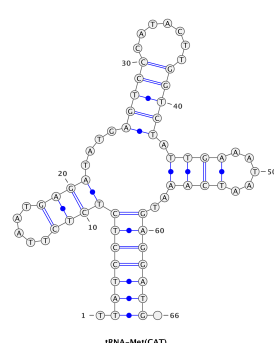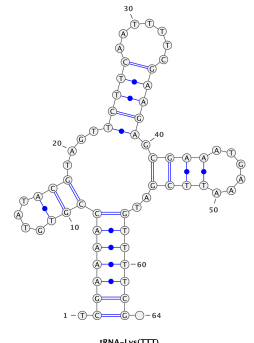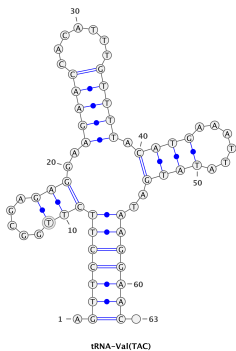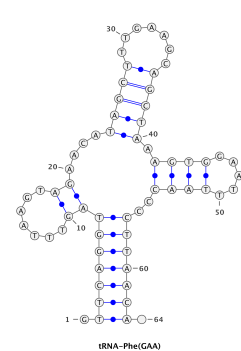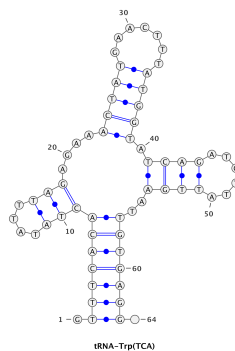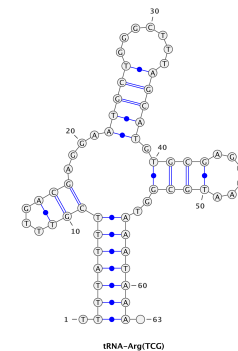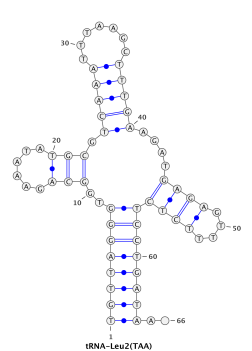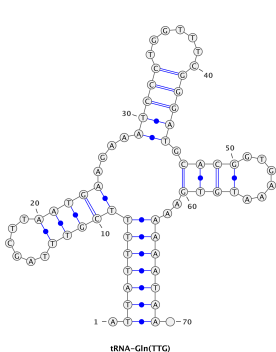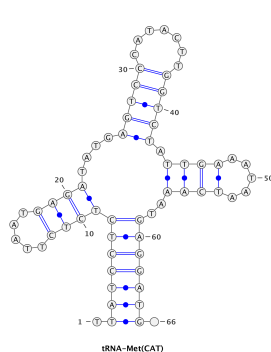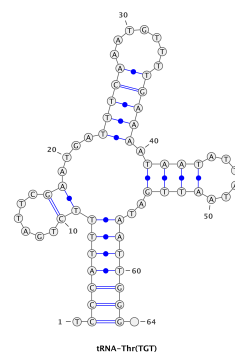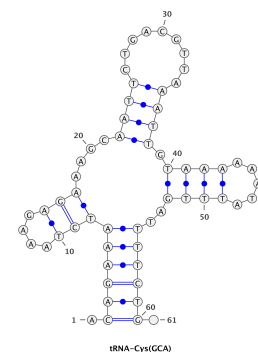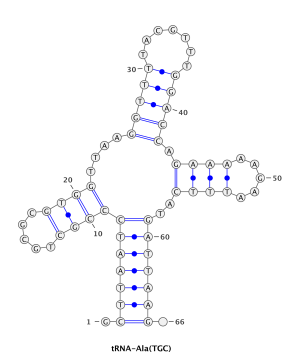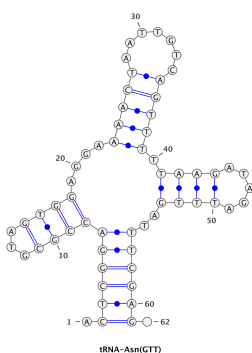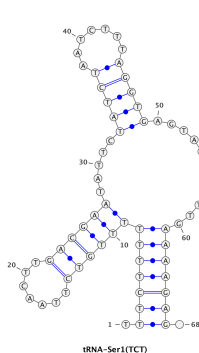

Supplement: Supplemental Information 1 [file peerj-05-3692-s001.pdf]
