# Supplementary material for: The complete mitochondrial genome of the grooved carpet shell, Ruditapes decussatus (Bivalvia, Veneridae)
Source: PeerJ. 2017 Aug 22;5:e3692. doi: 10.7717/peerj.3692 (PMC5571815; doi:10.7717/peerj.3692)
Supplement: Supplemental Information 2 [file peerj-05-3692-s002.pdf]

Name	Strand	Start	*p*-value		Sites	
PhUn	+	695	2.72e-19	AAAACTCAAA	GAGGGGGGGGGAAAGGGGGGGGGTCCCCAAA	AAATTCCTTT
MeLaM	+	539	1.87e-18	GGAGCAGGCT	TGGGGGGGGGGAAAAGGGGGGGGGGGAAAAA	AACAATAARA
RuPhM	+	3016	1.19e-17	GGAAAAATAC	TGTGGGGGGGGAAAGGGGGGGGGTCTCCAGA	AACTCCTTTC
RuPhF	+	3428	2.98e-17	GGACGTTGCT	ATGGGGGGGGGAAAGGGGGGGGGTCTCCAGA	AACTCCTTTC
MeLu	+	1230	5.38e-17	CAAGCTTAAA	TGGGGGGGGGGAAAATGGGGGGGGGGAAAAA	AGAAAATAAA
SeSc	+	82	9.47e-17	AGAAATGTGC	GTGGGGGGGGGAAGGGGGGGGGGGCCGTTAG	GCCGGAGAGG
MeMe	+	1240	1.88e-16	GCAAGCTTAA	GTGGGGGGGGGAAAATGGGGGGGGGCAGAAA	AATAGAAAAG
MePe	+	1240	4.68e-16	CAAGCTTAAG	TGGGGGGGGGGAAAATGGGGGGGGGGGGGGG	GGGCAGAAAA
MeLaF	+	558	4.68e-16	GGAGCAAGCT	TGGGGGGGGGGAAAAAGGGGGGGGGGAAAAA	GACGATTAAA
SoDi	+	34	1.26e-15	CTCCTCCTGT	AATGGGGGGGGGTAGGGGGGGGGTCGAAAAA	TGGAAGGAAA
PhTe	+	722	2.56e-15	TGTGTAGTTT	TTTGGGGGGGGAAAGGGGGGGGCACTAAAAA	AAATTCCTTT
StPu	+	72	4.05e-15	CTCTTTTGCA	TATGGGGGGGGGGGGGGGGGGGGACTCTAAA	TAATATATAA
PhEu	+	969	1.09e-14	AAGACGTTTC	TATGGGGGGGGAAAGGGGGGGGTCCTTAAAA	AAACTCCTTT
PhAm	+	2305	1.09e-14	TGTTTGTTTC	TATGGGGGGGGAAAGGGGGGGGCCCTTAAAA	AAACTCCTTT
MeLy	+	1159	2.27e-14	TGTGTATCTA	ATGGGGGGGGGTAAGGGGGGGGGTATAAAAG	TTGGTTATAT
RuDe	+	776	1.46e-13	ACAAACTACC	GTAAGGGGGGGATAAGGGGGGGTTCGCAAAA	AAACTCCTTT
SoDp	+	153	2.90e-12	AGTGGTGTTG	GATGTGGGGGGGGTAGGGGGGGGACCGAGCT	TCCGAAGGAA
SiCo	+	730	5.37e-12	ATATAAAATG	GAGCAGGGGGGAGGAGGGGAGGAGAGGAAAG	GAGCCCCTTT
FuMu	+	1386	7.13e-11	GGTATAAAAG	GTAAAGGGGGGAGAGGGGGAAGGGAGATTAA	ACCTCCCAAA
C0An	+	552	7.60e-11	GTGAAAAAAA	GGGGTGGGAGTAAAGGGGGGTCAGGGGCAGG	CAAGGCCCCT
AcTu	+	1007	2.18e-10	CCCCCCTTTC	TTTAAAGGGGGGGAGGGGGGGGTTCAATCCA	CCCCCCAAAG
MoIr	+	94	3.50e-10	AAGGAAAAAA	AGGGGGGGGGGGGTGTGGTATTCCGGAGT	AGAAGAGGAC
MoIr	+	94	3.50e-10	AAGGAAAAAA	AGGGGGGGGGGGGTGTGGTATTCCGGAGT	AGAAGAGGAC
NuOl	+	591	3.70e-10	CCCCTATGGG	GGGAGGGGGGGCCCGGGGAAAGGGCCCCCCC	TCCCCCCATA
LuRh	+	331	8.60e-10	TATTGAGGCA	GGGGAGGGAGGAGAATTTAGGGATCCGGGAG	GGATCAAAAA
