# Supplementary material for: The complete mitochondrial genome of the grooved carpet shell, Ruditapes decussatus (Bivalvia, Veneridae)
Source: PeerJ. 2017 Aug 22;5:e3692. doi: 10.7717/peerj.3692 (PMC5571815; doi:10.7717/peerj.3692)
Supplement: Supplemental Information 3 [file peerj-05-3692-s003.pdf]

Name	Strand	Start	*p*-value	Sites		
MeLy	+	2152	2.76e-18	ATACATTATT	GGGGGGGAGGGGGGTCTAAGGGGGGAGGG	GGGGGTTGCT
MePe	+	1717	1.31e-16	TCTTTTGTTA	GGGGGAGGGGGGGTTTAAGGGGGGGAGGG	GAATGTGATA
MeMe	+	1708	1.31e-16	TCTTTTGTTA	GGGGGAGGGGGGGTTTAAGGGGGGGAGGG	GAATATGATA
MeLu	+	1701	1.31e-16	TCTTTAATTA	GGGGGAGGGGGGGTTTAAGGGGGGGAGGG	GAATGTGATT
SiCo	+	1062	1.11e-14	TAGGTAAATT	GGGGGGAAGGGGGGTCCGGGGGGAAGGGG	GGGTAGCGTG
RuPhM	+	3514	2.27e-13	ATTAAAATGA	AAGAGGGGGGGGGGTCCTAGGGGGGGGGG	AGATTTTAGA
RuPhF	+	3929	5.15e-12	TGGTATATAA	GATTGGGGGGGGGGGTTTGGGGGGGGTTA	TTTAGAATTA
PhUn	+	495	1.82e-11	TGGGGTAAGG	GGTTGTAAGTGTAGTGTGGGCCGGGGGGG	ACCACACTAT
PhEu	+	740	1.82e-11	TAGGATAAAG	GATTGTAAGTGCGGTATAGGCCGGGGGGG	ACTATACCGT
MeLaM	+	1092	1.82e-11	TGTGTGTGTT	TGGGGGGAGAGGGGGAATGAGGGGGTTGG	CTATGGTTAG
MeLaF	+	1716	2.02e-11	ATAGAGGCTG	GGGGGGGTGGGGGTTTGTTGCAGTTAGGG	TATGGAAGTA
PhAm	+	2078	4.08e-11	TAGGATAAAG	GATTGTAAGTGCAGTGTGGGCCGGGGGGG	ACCACATTGT
SeSc	+	833	5.48e-11	CAGGATTTCC	TGGGGGGAGAGATTTCTGTGGGGGGGGGG	GTAAACGACA
PhTe	+	528	8.88e-11	TGGGAAAAAT	AGTTGTAAGTGCAGTATGGGCCGGGGGGG	ACTATATTGT
FuMu	-	1705	1.56e-10	GTATCCGGAA	GGGGGGAGGGGGGGAATTTGGGAGGTTTA	ATCTCCCTTC
SoDi	+	694	1.71e-10	ATTGATGTAG	GGTGGATAGTGGGGATGTGCTGGTGTGGG	GATAAATTAT
C0An	+	804	1.01e-09	GTTTAGTTGA	GTTTGGGATGGGGGTAAGAGGAAGAAGGG	GAAAGCAAGA
StPu	-	9	1.67e-09	CCCGAACGAC	GAAGGAGAGAGGGGTGGGAGCTGTGATGG	GGATTCTT
LuRh	+	747	2.13e-09	TTATGATAGA	GGGGGCGTGGGGGCTTATCCGGGTTATTG	TTGATAGGGT
MoIr	+	1344	6.40e-08	TGTTAATTTT	GGTTTGAATGGTATTGGGGGGAGTGTGTG	TAATTGTTTG
RuDe	-	1100	6.84e-08	CTCCATTTAC	GTTACGGAGGGGGCAAGTGCTGGGGTTTA	CTGGCGCCCG
NuOl	+	127	1.01e-07	TAAATTTAAT	TTGGCCGGGAGGAGAAAAAGGGGTAAGGC	AAACTAAAGT
SoDp	+	828	1.58e-07	TACTTGTGCT	ATGGTTTGGTGGATACTTGGTAGGAAGGG	ATACTCTTTT
AcTu	-	974	2.76e-07	TTTAAAGAAA	GGGGGGGAGGGGGAACTACCCCCCTGTTT	TTAGTGGCTT
