# Supplementary material for: The complete mitochondrial genome of the grooved carpet shell, Ruditapes decussatus (Bivalvia, Veneridae)
Source: PeerJ. 2017 Aug 22;5:e3692. doi: 10.7717/peerj.3692 (PMC5571815; doi:10.7717/peerj.3692)
Supplement: Supplemental Information 4 [file peerj-05-3692-s004.pdf]

Sample ID	Analysis	GenBank Accession	Data Availability
F3	Sanger sequencing of LUR	MF055702	GenBank
F4	Sanger sequencing of whole mitochondrial genome	KP089983	GenBank
F5	Sanger sequencing of LUR	MF055703	GenBank
F7	Sanger sequencing of LUR	MF055704	GenBank
F9	Sanger sequencing of LUR	MF055705	GenBank
F10	Sanger sequencing of LUR	MF055706	GenBank
F11	Sanger sequencing of LUR	MF055707	GenBank
F13	Sanger sequencing of LUR	MF055708	GenBank
F15	Sanger sequencing of LUR	MF055709	GenBank
F16	Sanger sequencing of LUR	MF055710	GenBank
F17	Sanger sequencing of LUR	MF055711	GenBank
F19	Sanger sequencing of LUR	MF055712	GenBank
F20	Sanger sequencing of LUR	MF055713	GenBank
F21	Sanger sequencing of LUR	MF055714	GenBank
fRDI01	*De novo* assembly of mtDNA; SNP	PRJNA170478	https://doi.org/10.6084/m9.figshare.4970762.v3; GenBank
fRDI02	*De novo* assembly of mtDNA; SNP	PRJNA170478	https://doi.org/10.6084/m9.figshare.4970762.v3; GenBank
fRDI03	*De novo* assembly of mtDNA; SNP	PRJNA170478	https://doi.org/10.6084/m9.figshare.4970762.v3; GenBank
fRDI04	*De novo* assembly of mtDNA; SNP	PRJNA170478	https://doi.org/10.6084/m9.figshare.4970762.v3; GenBank
fRDI05	*De novo* assembly of mtDNA; SNP	PRJNA170478	https://doi.org/10.6084/m9.figshare.4970762.v3; GenBank
fRDI06	*De novo* assembly of mtDNA; SNP	PRJNA170478	https://doi.org/10.6084/m9.figshare.4970762.v3; GenBank
mRDI01	*De novo* assembly of mtDNA; SNP	PRJNA170478	https://doi.org/10.6084/m9.figshare.4970762.v3; GenBank
mRDI02	*De novo* assembly of mtDNA; SNP	PRJNA170478	https://doi.org/10.6084/m9.figshare.4970762.v3; GenBank
mRDI03	*De novo* assembly of mtDNA; SNP	PRJNA170478	https://doi.org/10.6084/m9.figshare.4970762.v3; GenBank
mRDI04	*De novo* assembly of mtDNA; SNP	PRJNA170478	https://doi.org/10.6084/m9.figshare.4970762.v3; GenBank
mRDI05	*De novo* assembly of mtDNA; SNP	PRJNA170478	https://doi.org/10.6084/m9.figshare.4970762.v3; GenBank
mRDI06	*De novo* assembly of mtDNA; SNP	PRJNA170478	https://doi.org/10.6084/m9.figshare.4970762.v3; GenBank
