# Supplementary material for: The complete mitochondrial genome of the grooved carpet shell, Ruditapes decussatus (Bivalvia, Veneridae)
Source: PeerJ. 2017 Aug 22;5:e3692. doi: 10.7717/peerj.3692 (PMC5571815; doi:10.7717/peerj.3692)
Supplement: Supplemental Information 5 [file peerj-05-3692-s005.pdf]

Primer	Direction	Sequence 5'-3'	amplified	Lenght(bp)
ATP6-1	F	ATTTGTGCTTCTTTTCAGTGTACTTTT	1517 bp	27
ATP6-1	R	TCATCCCAATTACTAGAATAAAACAGG		27
ATP6-3	F	TAACTGGTTGGTTCCCTTAATGTT	1321 bp	24
ATP6-3	R	AACTTTAATAGGGGAGTTCTTCG		23
ATP6-4	F	TTAAGGAAACGGTGTCTTATTTTTG	1379 bp	25
ATP6-4	R	CTAGGAAAACAGATCACTGACAAGA		25
ATP6-5	F	TTAGTTTGGTTGGGGTAATAATAAGG	1479 bp	26
ATP6-5	R	GTTAGCACAAATACAACTACCACCAT		26
ATP6-6	F	TATAGATTGAAATCAGTTGGGTTTTTC	1259 bp	27
ATP6-6	R	TCAACTACCCTAGATATCCTTTACTGC		27
CYTB-1	F	GATCATATAAACCGTAAGCGAATAATG	1433 bp	27
CYTB-1	R	ATATCTAGCAACTAACCCAACCCTAAT		27
ATP8-2	F	GGGCGTGTAGATGAATAGATTTTTA	1255 bp	26
ATP8-2	R	CTCTAAAGAAGGGATCACACCATAA		26
ND6-1	F	TAAATGTAATAAGGCGCTATAATCACC	1242 bp	28
ND6-1	R	TATTTCTGCCACCCTAACAAAATAAAG		27
COX3-2	F	TTAAATTAACCTTAACCAACGAAATAGG	1285 bp	29
COX3-2	R	TAATTCGAATTTGCTTTTTATTAAGTGA		29
ATP8-ND6	F	GCTTTAGGGTGTTCGATTCACTCAGGA	1323 bp	27
ATP8-ND6	R	AAACGCCCCCGTAAAAGCTAAGAACAC		27
ATP6-CYTB	F	AGTTGTGTGGATCTGGCCACAGAGAAA	1300 bp	27
ATP6-CYTB	R	TACCTGGGAAACCCCCATTATTCGCTT		27
ND6COX3	F	ATGGCGTAATGGAGGGTGTACGATTTC	1218 bp	27
COX3ATP6	R	CACCGGCCATTTAGAAATTTCAGGCA		26
CYTB-2	F	TCTGTGTTCAAAAATGTACAGCATAAT	1318 bp	27
CYTB-2	R	CTTTCTTTTTCAGAGACAAGCAACTT		26
ATP6-2R	F	ATTAATAGTAGGTTGGGATGGTTTAGG	1501 bp	27
ND6COX3-R1	R	AAATCGCCCGGCGTTCTTGAATACTGA		27
ATP62R	F	TTCATGTAATTTTAGGAATTTGCTTTC	2283 bp	27
COX3-R1	R	AAATCGCCCGGCGTTCTTCTTGAATACTGA		30
ATP6-3R	F	AACTTTAATAGGGGAGTTCTTCG	1729 bp	23
ND6COX3-R1	R	AAATCGCCCGGCGTTCTTGAATACTGA		28
ATP6-3R	F	AACTTTAATAGGGGAGTTCTTCG	2028 bp	23
COX3-R1	R	TTCATGTAATTTTAGGAATTTGCTTTC		27

ATP6CYTB-F	F	AGTTGTGTGGATCTGGCCACAGAGAAA	1418 bp	28
CYB-R1	R	ATATCTAGCAACTAACCCAACCCTAAT		28
ATP6-5	F	TTAGTTTGGTTGGGGTAATAATAAGG	2076 bp	26
ATP6-6	R	TCAACTACCCTAGATATCCTTTACTGC		28
NewATP8	F	AAGGGGAGGTAGCGAGAAAA	729 bp	20
NewND4	R	GGCAACGAGGAACCTACAGT		20
New16S	F	GTGACACGGTGGATTATTGCTT	1256 bp	22
NewATP6	R	AAACCCCATCACACAAACACAC		22
NewCYTB1	F	TGGGTACATGTCCCGTAGAAGA	870 bp	22
New16S	R	TATGAACGCCTTACCCTATCCC		22
NewND5	F	TGGGGCTCTTTCAATAGCTGT	1731 bp	21
NewATP8	R	GCAAGCAAGAGGAGCAAACT		20
NewCR-F3	F	TGTAGAAATAGGCTGAATTCGAGG	1188 bp	24
NewCR-F4	R	ATAACTTTTGCGGCCCTTAGTC		22
NewCR-F4	F	TTGTGATAACTGCTAGGGTGGT	1529 bp	22
