# Supplementary material for: The complete mitochondrial genome of the grooved carpet shell, Ruditapes decussatus (Bivalvia, Veneridae)
Source: PeerJ. 2017 Aug 22;5:e3692. doi: 10.7717/peerj.3692 (PMC5571815; doi:10.7717/peerj.3692)
Supplement: Supplemental Information 6 [file peerj-05-3692-s006.pdf]

Species	GenBank Acc. No.	BRIGS	Easyfig	MEME	MitoPhast
Acanthocardia tuberculata	NC_008452			x	
Arctica islandica	NC_022709			x	x
Coelomactra antiquata	NC_021375			x	
Fulvia mutica	NC_022194			x	
Hiatella arctica	NC_008451			x	
Loripes lacteus	NC_013271			x	
Lucinella divaricata	NC_013275			x	
Lutraria rhynchaena	NC_023384			x	
Meretrix lamarckii	NC_016174	x	x		
Meretrix lamarckii F-type	KP244451			x	x
Meretrix lamarckii M-type	KP244452			x	x
Meretrix lusoria	NC_014809	x		x	x
Meretrix meretrix	NC_013188	x		x	x
Meretrix petechialis	NC_012767	x		x	x
Moerella iridescens	NC_018371			x	
Nuttallia olivacea	NC_018373			x	
Paphia amabilis	NC_016889	x	x	x	x
Paphia euglypta	NC_014579	x		x	x
Paphia textile	NC_016890	x		x	x
Paphia undulata	NC_016891	x		x	x
Ruditapes philippinarum F-type	AB065375	x	x	x	x
Ruditapes philippinarum M-type	AB065374	x		x	x
Semele scabra	NC_018374			x	
Sinonovacula constricta	NC_011075			x	
Solecurtus divaricatus	NC_018376			x	
Solen grandis	NC_016665			x	
Solen strictus	NC_017616			x	
Soletellina diphos	NC_018372			x	
Strongylocentrotus purpuratus	NC_001453			x	
