# Supplementary material for: The complete mitochondrial genome of the grooved carpet shell, Ruditapes decussatus (Bivalvia, Veneridae)
Source: PeerJ. 2017 Aug 22;5:e3692. doi: 10.7717/peerj.3692 (PMC5571815; doi:10.7717/peerj.3692)
Supplement: Supplemental Information 7 — BP, Biological Process; CC, Cellular Component; MF, Molecular Function. [file peerj-05-3692-s007.pdf]

**Supplementary Table 4** Most significant GO terms associated with the two DNA motifs found in the LUR. BP = Biological Process; CC = Cellular Component; MF = Molecular Function.

| Motif 1 | Motif 2 |
|---|---|
| Positive regulation of transcription from RNA polymerase II promoter (BP) | Transcription (BP) |
| Transcription (BP) | Negative regulation of transcription from RNA polymerase II promoter (BP) |
| Negative regulation of transcription from RNA Polymerase II promoter (BP) | - |
| Transcription factor complex (CC) | - |
| Transcription activator activity (MF) | - |
